# Durable Response to Selpercatinib in Metastatic Colorectal Cancer Harboring a Novel *TIMM23B::RET* Fusion: A Case Report

**DOI:** 10.3390/curroncol33050271

**Published:** 2026-05-07

**Authors:** Ziyan Tong, Mengyuan Yang, Shanshan Weng, Ying Yuan

**Affiliations:** 1 Department of Medical Oncology, Key Laboratory of Cancer Prevention and Intervention, Ministry of Education, The Second Affiliated Hospital, Zhejiang University School of Medicine, Hangzhou 310009, China; 2 Zhejiang Provincial Clinical Research Center for CANCER, Hangzhou 310009, China; 3 Cancer Center, Zhejiang University, Hangzhou 310058, China; 4 Center for Medical Research and Innovation in Digestive System Tumors, Ministry of Education, Hangzhou 310009, China

**Keywords:** metastatic colorectal cancer, RET fusion, TIMM23B::RET, selpercatinib, precision oncology, case report

## Abstract

*RET* fusions are rare but actionable oncogenic drivers in metastatic colorectal cancer (mCRC), occurring in a small molecular subset with limited clinical evidence for selective RET inhibition. We report a 77-year-old woman with mCRC harboring a rare *TIMM23B::RET* fusion who achieved durable benefit from selpercatinib after progression on capecitabine. Molecular profiling showed *RAS/BRAF* wild-type, microsatellite-stable disease with a *TIMM23B::RET* fusion. Selpercatinib induced a marked decline in tumor markers and a sustained partial response on serial imaging. Treatment was complicated by grade 3 hepatotoxicity, requiring temporary interruption and dose reduction to 80 mg twice daily. Despite this, disease control was maintained without further grade ≥ 3 toxicity. At the time of writing, the patient remains on treatment with progression-free survival exceeding 14 months. To our knowledge, this is among the first detailed reports of mCRC harboring a *TIMM23B::RET* fusion with documented clinical benefit from selpercatinib. This case highlights the value of comprehensive genomic profiling and individualized toxicity management in rare molecular subsets of mCRC.

## Simple Summary

Metastatic colorectal cancer is usually treated according to common molecular alterations, but rare gene fusions may also provide therapeutic opportunities. We describe a patient with metastatic colorectal cancer harboring a rare *TIMM23B::RET* fusion who achieved a durable response to selpercatinib after progression on capecitabine. Treatment produced a marked decline in tumor markers and a sustained partial radiographic response. Although grade 3 hepatotoxicity occurred, the patient was able to continue treatment after temporary interruption and dose reduction, with disease control maintained for more than 14 months. This case highlights the importance of comprehensive genomic profiling in metastatic colorectal cancer and suggests that careful management of adverse events may help maintain the benefit of targeted therapy.

## 1. Introduction

Colorectal cancer (CRC) remains a leading cause of cancer-related morbidity and mortality worldwide [1]. With the advancements in precision medicine, molecular targeted therapies have emerged as a critical tool for personalized CRC management. Among these, rearranged during transfection (*RET*) gene fusion represents an emerging yet rare therapeutic target. *RET* fusions are most prevalent in thyroid cancer (5–10%) and non-small cell lung cancer (NSCLC) (1–2%), occurring in less than 1% of other solid tumors, including CRC [2,3]. Studies indicate that *RET* fusions predominantly occur in elderly patients with Eastern Cooperative Oncology Group performance status (ECOG PS) 1–2, right-sided colon tumors with *RAS/BRAF* wild-type and MSI-H phenotypes, accounting for approximately 0.4% of metastatic CRC (mCRC) cases [4,5]. Additionally, this rare subgroup of patients tends to exhibit poorer outcomes, with a median overall survival (OS) of only 14.0 months [6]. Thus, developing precision-targeted therapies is essential for this rare subgroup of CRC patients.

**Figure 1:**
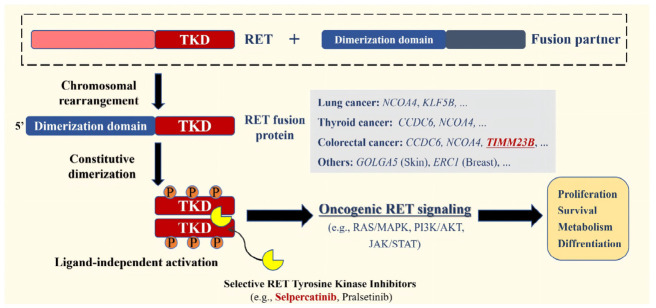
Oncogenic mechanism and targeted therapy of *RET* fusions driven by chromosomal rearrangements**.** *RET* fusion proteins retain the intracellular tyrosine kinase domain and can undergo ligand-independent activation, resulting in constitutive downstream signaling through pathways such as MAPK and PI3K-AKT-mTOR. Selective RET inhibitors, including selpercatinib and pralsetinib, are designed to block this aberrant signaling. Abbreviations: RET, rearranged during transfection; TKD, tyrosine kinase domain.

The *RET* proto-oncogene encodes a transmembrane glycoprotein receptor tyrosine kinase (RTK). Aberrant RET activation often leads to constitutive stimulation of downstream signaling pathways such as MAPK, PI3K-AKT-mTOR, thereby driving tumor proliferation and metastasis [7]. Small-molecule RET-specific tyrosine kinase inhibitors (TKIs) have been developed, including selpercatinib and pralsetinib (Figure 1). Until now, selpercatinib has demonstrated significant efficacy in patients with *RET* fusion-positive non-small cell lung cancer (NSCLC) [8,9]. However, its therapeutic potential in other advanced solid tumors, including CRC, remains under investigation. One available source of clinical evidence is the LIBRETTO-001 study (NCT03157128). In this basket trial, 10 *RET* fusion-positive mCRC patients showed an objective response rate (ORR) of 20% and a median duration of response of 9.4 months [10]. This indicates that the current clinical data of selpercatinib in mCRC remain insufficient to establish definitive conclusions, and the therapeutic relevance of uncommon *RET* fusion partners remains largely undefined. Selpercatinib has received FDA approval for treating *RET* fusion-positive advanced solid tumors and has been included in the 2024 NCCN (National Comprehensive Cancer Network) Clinical Practice Guidelines for CRC [11,12,13]. Nevertheless, additional evidence from large-scale phase III clinical trials is needed to validate the efficacy data of selpercatinib in mCRC.

In clinical practice, the interpretation of *RET* rearrangements in CRC remains challenging because both the prevalence and partner spectrum are limited, and therapeutic evidence is still largely derived from basket trials and isolated case reports. This creates uncertainty not only in treatment selection but also in the biologic interpretation of rare *RET* fusion partners detected by routine genomic profiling. Accordingly, detailed clinicopathologic case descriptions remain valuable, particularly when they include molecular context, treatment course, toxicity management, and durable follow-up. Herein, we present a case of mCRC harboring a rare *TIMM23B::RET* fusion, in which the patient derived significant and durable clinical benefit from selpercatinib. *TIMM23B::RET* has been reported as an uncommon *RET* fusion event in sequencing-based studies of solid tumors [14,15], but its clinical relevance remains poorly defined, particularly in CRC. This case expands the clinical spectrum of *RET*-rearranged CRC and underscores the importance of comprehensive genomic profiling and individualized toxicity management.

## 2. Case Presentation

### 2.1. Initial Presentation and Diagnostic Workup

A 77-year-old woman presented with markedly elevated tumor markers, including carcinoembryonic antigen (CEA) at 41 ng/mL and carbohydrate antigen 19-9 (CA19-9) at 3400 U/mL. She had a history of hypertension and was receiving metoprolol and amlodipine as chronic medication. She had no history of diabetes mellitus, arrhythmia, hepatitis, chronic kidney disease, or other major comorbidities. Baseline laboratory evaluation before systemic treatment showed normal liver and renal function, including alanine aminotransferase (ALT), aspartate aminotransferase (AST), total bilirubin, serum creatinine, and estimated glomerular filtration rate (eGFR). Her ECOG PS was 2.

**Figure 2:**
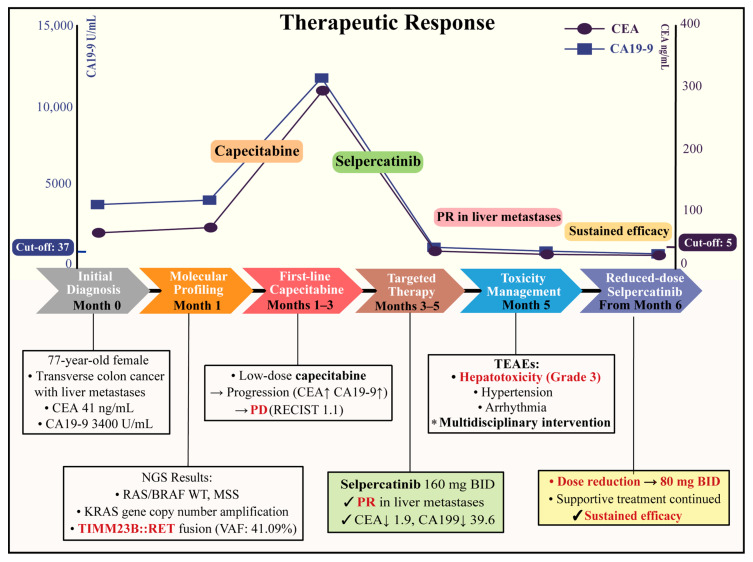
Timeline of clinical course, biomarker dynamics, treatment modifications, and therapeutic response. The figure summarizes the patient’s clinical course from initial diagnosis through first-line capecitabine treatment, molecular profiling, selpercatinib therapy, toxicity management, and adapted low-dose treatment. After progression on low-dose capecitabine, selpercatinib was associated with a marked decline in serum CEA and CA19-9 levels and a partial response in liver metastases. Grade 3 hepatotoxicity and other treatment-related adverse events were managed with temporary interruption, supportive care, and dose reduction, after which sustained clinical benefit was maintained. Dashed horizontal lines indicate the upper limits of normal for CA19-9 (37 U/mL) and CEA (5 ng/mL). Abbreviations: CEA, carcinoembryonic antigen; CA19-9, carbohydrate antigen 19-9; MSS, microsatellite stable; NGS, next-generation sequencing; RET, rearranged during transfection; TEAEs, Treatment-related adverse events; PR, partial response; PD, progressive disease; RECIST, Response Evaluation Criteria in Solid Tumors.

Systemic positron emission tomography-computed tomography and colonoscopy confirmed the diagnosis of transverse colon cancer with multiple liver metastases. Histopathological examination of the colonoscopic biopsy showed poorly differentiated adenocarcinoma with neuroendocrine differentiation. Immunohistochemistry demonstrated positive expression of CDX2, SATB2, and CK7, focal positivity for Synaptophysin, Chromogranin A, and INSM1, and negative expression for HepPar1, Arginase-1, p40, POU2F3, and CD56. Mismatch repair proficiency (pMMR) was also confirmed. Collectively, the integrated clinicopathological findings supported the diagnosis of primary metastatic colorectal cancer (Figure 2).

### 2.2. Molecular Findings and First-Line Treatment

Targeted next-generation sequencing (NGS) was performed on formalin-fixed, paraffin-embedded (FFPE) unstained slides prepared from the colonoscopic biopsy specimen using a 425-gene solid tumor panel with hybrid-capture-based high-throughput sequencing. The sample passed quality control, with a tumor cell content of 50%, an average sequencing depth of 2420.12×, and an effective sequencing depth of 2000.57×. Molecular profiling identified no *KRAS/NRAS/BRAF* hotspot activating mutations, microsatellite stability (*MSS*), low tumor mutational burden (4.1 mutations/Mb), *KRAS* copy number amplification (copy number 5.22), and a *RET* rearrangement identified as *TIMM23B::RET* (*TIMM23B* exon 6—*RET* exon 12) with a variant allele frequency of 41.09%.

Given her advanced age, ECOG PS of 2, and reluctance to receive intravenous anticancer therapy, low-dose capecitabine was initiated as first-line treatment. However, after 9 weeks (3 cycles), tumor markers increased markedly (CEA 286 ng/mL; CA19-9 > 12,000 U/mL; Figure 2), accompanied by intolerable grade 2 nausea and vomiting. Follow-up contrast-enhanced abdominal computed tomography demonstrated enlargement of the liver metastases, consistent with progressive disease according to RECIST version 1.1.

### 2.3. Selpercatinib Treatment and Tumor Response

**Figure 3:**
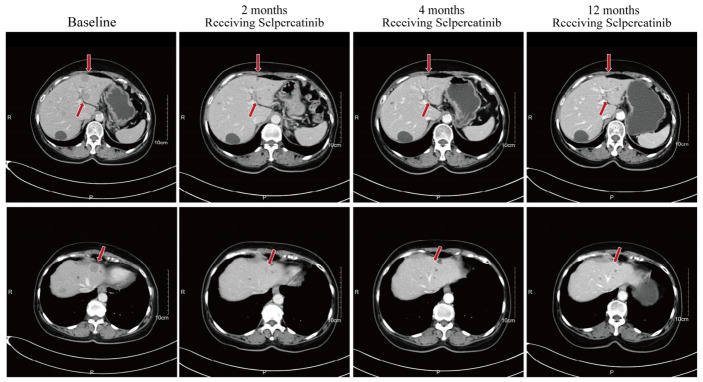
Serial contrast-enhanced abdominal computed tomography showing partial response of hepatic metastases during selpercatinib treatment. Representative axial CT images obtained at baseline and during follow-up after selpercatinib initiation demonstrate a marked reduction in the size of hepatic metastatic lesions over time, consistent with partial response according to RECIST version 1.1. Red arrows indicate representative hepatic target lesions. The upper and lower rows show two representative axial levels. Abbreviations: CT, computed tomography; PR, partial response; RECIST, Response Evaluation Criteria in Solid Tumors.

Then, based on the identified *TIMM23B::RET* fusion, selpercatinib was initiated at 160 mg twice daily after informed consent (Figure 2). Following treatment initiation, serum tumor markers declined substantially, reaching a nadir of 1.6 ng/mL for CEA and 10.6 U/mL for CA19-9. Serial follow-up imaging at 2, 4, 6, and 12 months revealed a substantial partial response in liver metastases (Figure 3) and primary tumor.

### 2.4. Adverse Events and Dose Modification

Treatment-related adverse events (AEs), graded according to CTCAE version 5.0, are summarized in Table 1. Early after treatment initiation, the patient developed several toxicities, including grade 3 hepatotoxicity, grade 2 hypertension, grade 2 arrhythmia, grade 1 abdominal distension, and grade 2 xerostomia. These events were managed with supportive care and appropriate symptomatic treatment, including antihypertensive medication and cardiology-guided management when indicated. The most clinically significant toxicity was hepatotoxicity, with ALT peaking at 1142 U/L and AST at 908 U/L, respectively, accompanied by malaise. Selpercatinib was therefore temporarily interrupted, and hepatoprotective therapy was administered with close laboratory monitoring. After liver function improved, treatment was resumed at a reduced dose of 80 mg twice daily. Subsequently, no further grade ≥ 3 adverse events were observed. Mild peripheral edema developed later during treatment and improved with diuretic therapy. Overall, as of the writing of this paper, the patient has remained on low-dose selpercatinib with tolerable toxicity and progression-free survival exceeding 14 months.

## 3. Discussion

This case provides clinically relevant evidence supporting the use of selective RET inhibition in a rare molecular subset of mCRC. *RET* fusions are uncommon in CRC, and currently available efficacy data for selpercatinib in this population remain limited. In contrast to the modest activity observed in small basket trial cohorts [10], our patient experienced a deep and durable response, supporting the view that *RET* rearrangements may represent clinically actionable targets in selected cases of mCRC.

A key strength of this report is the identification of a rare *TIMM23B::RET* fusion in CRC. While *KIF5B*, *CCDC6*, *NCOA4*, and other *RET* fusion partners have been described across solid tumors [4], available data suggest that *RET*-rearranged CRC represents a distinct molecular subset in which the spectrum of fusion partners is broader than the few recurrent partners most commonly emphasized in other tumor types. In this context, *TIMM23B::RET* appears to represent a particularly rare partner, for which clinical evidence remains extremely limited. Previous sequencing studies have identified *TIMM23B* as a rare *RET* fusion partner in solid tumors [14,15], but clinical data remain extremely limited. *TIMM23B* encodes a component of the mitochondrial inner membrane translocase complex, although its specific contribution as a *RET* fusion partner has not been well defined [16]. In this case, the rearrangement involved *TIMM23B* exon 6 and *RET* exon 12, preserving the RET kinase domain, which is the principal actionable region targeted by selective RET inhibitors (Figure 1). This structural feature provides a biologically plausible explanation for the observed sensitivity to selpercatinib. However, whether *TIMM23B* contributes a functionally relevant dimerization motif, altered expression context, or other oncogenic property remains uncertain. Compared with more established *RET* fusion partners, the therapeutic significance of rare partners such as *TIMM23B* remains less well defined. Our case suggests that when the RET kinase domain is preserved, selective RET inhibition may still provide clinically meaningful benefit, although broader generalization is not yet possible. Together with the limited published evidence, including basket-trial data [8] and an individual case report of mCRC harboring *NCOA4-RET* treated with selpercatinib [17], this case adds to the emerging evidence that rare *RET*-rearranged CRC may still derive benefit from selective RET inhibition. Notably, *NCOA4* has been reported as the most common *RET* fusion partner in CRC [18], whereas *TIMM23B::RET* appears to be considerably rarer. In this context, our case extends the clinical spectrum of selpercatinib-responsive *RET*-rearranged CRC from a more established fusion partner to a rare and much less well-characterized partner. Therefore, the clinical response observed here should be interpreted as supportive, but not definitive, evidence that *TIMM23B::RET* may represent a biologically relevant and therapeutically targetable alteration in this case.

In addition, the biopsy showed focal neuroendocrine marker expression. However, when interpreted in the context of the overall morphologic and immunophenotypic findings, the tumor was considered colorectal adenocarcinoma with neuroendocrine differentiation rather than a mixed neuroendocrine-non-neuroendocrine neoplasm or pure neuroendocrine carcinoma. Recent pathologic evidence suggests that colorectal adenocarcinomas with limited neuroendocrine components should be interpreted cautiously and are biologically distinct from bona fide mixed neuroendocrine tumors [19]. In this case, neuroendocrine differentiation was regarded as a noteworthy pathologic feature, but it did not independently alter treatment selection. Concurrent *KRAS* amplification was also identified in this case and may be biologically relevant. Recent evidence in CRC suggests that *KRAS* amplification, particularly in *KRAS* wild-type tumors, may be associated with adverse clinicopathological features, unfavorable prognosis, increased downstream pathway activation, and reduced sensitivity to anti-EGFR therapy in some settings [20]. Similar observations from other tumor types further support the potential biologic relevance of *KRAS* copy number gain [21]. However, its functional and therapeutic significance in *RET* fusion-positive CRC remains unclear. Notably, in our patient, the presence of *KRAS* amplification did not preclude a deep and durable response to selpercatinib, suggesting that the predictive value of this co-alteration in this context should be interpreted cautiously. Overall, these findings suggest that some rare *RET* fusion partners may warrant consideration as clinically relevant targets for selective RET inhibition.

Another important aspect of this case is the management of treatment-related toxicity. Achieving an optimal balance between therapeutic efficacy and adverse reactions remains a critical challenge in clinical practice. In the phase III LIBRETTO-431 trial (NCT04194944) involving NSCLC patients, grade ≥ 3 adverse events of selpercatinib included hypertension (20%), aspartate (13%), and alanine aminotransferase elevation (22%) [8]. Similarly, updated LIBRETTO-001 trial data revealed hypertension (19.3%) and the elevations of transaminase (aspartate aminotransferase: 10.2%; alanine aminotransferase: 14.9%) as predominant grade ≥ 3 adverse events in patients with *RET* fusion-positive solid tumors [9]. In this case, the patient developed grade 3 hepatotoxicity and multiple additional adverse events during selpercatinib treatment. Nevertheless, prompt interruption, multidisciplinary supportive care, and cautious reintroduction at a reduced dose enabled continued therapy without loss of clinical benefit. This observation is clinically meaningful because it suggests that treatment efficacy may be preserved despite substantial dose reduction [22], provided that adverse events are recognized early and managed proactively.

From a broader clinical perspective, this case has several potential implications. First, it supports the value of comprehensive genomic profiling in selected patients with mCRC, particularly when standard treatment options are limited or when the clinical course appears atypical. Second, it suggests that rare *RET* fusion partners, even when not among the best-characterized canonical rearrangements, may still warrant therapeutic consideration if the RET kinase domain is preserved. Third, it highlights the need for future multicenter registries and molecularly annotated case series to better define the prevalence, biologic significance, and treatment responsiveness of uncommon *RET* fusion partners in CRC. Where feasible, orthogonal molecular confirmation and functional studies will be important to distinguish biologically relevant rearrangements from incidental findings and to refine patient selection for selective RET inhibition.

Taken together, this case expands the clinical spectrum of *RET*-rearranged mCRC and adds to the limited evidence supporting selpercatinib in this setting [10], particularly in tumors harboring rare *RET* fusion partners. It also provides practical insight into individualized precision therapy in an elderly patient, highlighting sustained clinical benefit despite dose reduction and emphasizing the importance of vigilant monitoring for treatment-related toxicities, particularly severe hepatotoxicity requiring temporary interruption. In addition, this case supports the value of broad genomic testing in refractory or atypical mCRC to identify rare actionable drivers [23]. However, several limitations should be acknowledged. This is a single-case report, and the *TIMM23B::RET* rearrangement was identified by a clinically validated DNA-based targeted NGS assay without orthogonal confirmation by RNA sequencing, RT-PCR, or FISH. In addition, no functional experiments were performed to establish the oncogenic role of this fusion, and a direct causal link between *TIMM23B::RET* and the observed therapeutic response cannot be established from this single case alone. Therefore, the findings should be interpreted as hypothesis-generating. Further studies are warranted to clarify the biologic significance of rare *RET* fusion partners in CRC, validate the role of RET inhibitors in mCRC, and refine toxicity management strategies.

## 4. Conclusions

We report a rare case of metastatic colorectal cancer harboring a *TIMM23B::RET* fusion that achieved a durable response to selpercatinib. This case broadens the molecular and clinical landscape of *RET*-rearranged CRC and supports the use of comprehensive genomic profiling to identify rare actionable alterations. It also illustrates that durable benefit may be maintained despite dose reduction when treatment-related toxicities are managed promptly and individually. More broadly, this case underscores the need for continued accumulation of clinically annotated evidence to refine the interpretation and therapeutic relevance of rare *RET* fusion partners in CRC.

## Figures and Tables

**Table 1:** Treatment-related adverse events during selpercatinib therapy.

Adverse Event	Maximum CTCAE Grade	Time of Onset After Selpercatinib Initiation *	Management	Outcome
Hepatotoxicity	3	During the early treatment period (Month 1)	Temporary interruption; intravenous and oral hepatoprotective treatment; close laboratory monitoring	Improved; no recurrent grade ≥ 3 event
Hypertension	2	Early after initiation (Month 1)	Cardiology consultation; antihypertensive treatment	Controlled
Arrhythmia	2	Early after initiation (Month 1)	Rate-control medication and cardiology follow-up	Improved
Xerostomia	2	Early after initiation (Month 1)	Supportive care and oral hydration	Improved after dose adjustment
Abdominal distension	1	Early after initiation (Month 1)	Supportive care and observation	Improved
Peripheral edema	1	During ongoing low-dose treatment (Month 3)	Diuretics and supportive care	Improved

* Note: Time of onset was calculated from selpercatinib initiation. Abbreviations: CTCAE, Common Terminology Criteria for Adverse Events.

## Data Availability

The original contributions presented in this study are included in the article. Further inquiries can be directed to the corresponding authors.

## Abbreviations

AE, adverse event; ALT, alanine aminotransferase; AST, aspartate aminotransferase; BID, twice daily; CA19-9, carbohydrate antigen 19-9; CEA, carcinoembryonic antigen; CRC, colorectal cancer; CT, computed tomography; CTCAE, Common Terminology Criteria for Adverse Events; ECOG PS, Eastern Cooperative Oncology Group performance status; eGFR, estimated glomerular filtration rate; FFPE, formalin-fixed, paraffin-embedded; mCRC, metastatic colorectal cancer; MSS, microsatellite stable; NGS, next-generation sequencing; NSCLC, non-small cell lung cancer; ORR, objective response rate; OS, overall survival; PD, progressive disease; pMMR, proficient mismatch repair; PR, partial response; RECIST, Response Evaluation Criteria in Solid Tumors; RET, rearranged during transfection; RTK, receptor tyrosine kinase; RT-PCR, reverse transcription polymerase chain reaction; TEAEs, treatment-related adverse events; TKI, tyrosine kinase inhibitor; TKD, tyrosine kinase domain.
